# Perceptions of health among school-aged children living in socially vulnerable areas in Sweden

**DOI:** 10.3389/fpubh.2023.1136832

**Published:** 2023-07-07

**Authors:** Gabriella E. Isma, Margareta Rämgård, Karin Enskär

**Affiliations:** ^1^Department of Care Science, Faculty of Health and Society, Malmö University, Malmö, Sweden; ^2^Research Program: Equal Health – Health Promotion Platform in Collaboration, Malmö University, Malmö, Sweden; ^3^Department of Women’s and Children’s Health, Uppsala University, Uppsala, Sweden

**Keywords:** multi-activity center, health, well-being, participatory design, schoolchildren, socially vulnerable area

## Abstract

**Introduction:**

According to the Convention on the Rights of the Child, all children have the right to health. Since good health is a decisive factor for children’s future, investing in children’s health is important, especially children from vulnerable areas. The purpose of this study was to investigate the perceptions of health among school-aged children from socially vulnerable areas.

**Methods:**

The study has an explorative mixed-method design with a participatory and inductive approach based on focus group interviews with children and youth leaders, respectively, at Multi-activity Centers in three of the vulnerable areas in Malmö Municipality, as well as results from the Multi-activity Centers’ own questionnaire. The data has been analyzed with inductive and deductive content analysis.

**Results:**

The children and the youth leaders described health in terms of well-being, participation, and activity. Well-being included feeling good and safe, having a healthy body, and having fun by doing things together with friends and leaders. Participating in activities was described as having a feeling of involvement, being able to have an influence on the organization of the activities and participating on one’s own terms.

**Discussion:**

The result of this study shows that participating in activities increases the child’s sense of well-being.

## Introduction

Children face many pressures and challenges. The social environment of children living in deprived city areas, and having fewer social resources, may easily influence their health and health-related behavior. Furthermore, childhood behaviors can continue into adulthood, affecting issues such as mental health, the development of health complaints, tobacco and alcohol use, diet, and physical activity level.

There is a growing concern in society for the health and well-being of children. During childhood, an individual acquires the physical, cognitive, emotional, social, and economic resources that are the foundation for health and well-being later in life. These same resources define the transition into the next generation. Investments in child health and well-being will bring benefits today, for decades to come, and for the next generation (1). The cross-national study *Health Behavior of School-aged Children* (HBSC) has noted the importance of the social context and children’s relations with families, peers, and school. The study concludes that a supportive environment could play an important role for a healthy development (2). In Sweden, a new concept called “Allaktivitetshus,” that is, Multi-activity Centers (MaCs), has been developed to promote a healthier lifestyle through activities for children and young adults living in the city.

In 1989, the General Assembly of the United Nations adopted and opened the resolution about the *Convention on the Rights of the Child* (3, 4). Sweden was one of the first states in the world to ratify the convention in 1990, but it came into force rather late, in January 2020 (5). The convention states that all children, without exception, have all the rights listed in the convention. Sweden has been criticized by (6), an international non-governmental organization, for implementing children’s rights inconsistently. Some researchers also argue that discrimination against children has increased (7). This will probably affect children’s development and health.

According to the World Health Organization (8), *health* is “a state of complete physical, mental and social well-being, not just the absence of illness and disability.” Earlier research has shown that age is also an important factor in the understanding of the concept of health (9–11). Therefore, understanding variations of health perceptions related to age is important for researchers and professionals involved in health promotion and interventions directed toward children.

For young children, participation is mostly about play and activity together with significant others in everyday situations (12). The importance for children of participating in meaningful *activities*, is stated by the UNCRC (3), and such activities have several positive effects on children’s health and development (13). School-aged children have described leisure activities as joint and meaningful activities (14) and participating in activities has often been related to health and security for children (15). Previous research also shows that children’s involvement in organized activities can be linked to their engagement in social relationships, their achievement in school, and their satisfaction in life (16–18). Children living in vulnerable areas have been shown to be less engaged in organized activities (19), and evidence demonstrates that disadvantaged social circumstances are associated with increased health risks (20–22). However, little attention has been paid to inequalities related to socioeconomic status, age, and gender among children (23).

The *Multi-activity Centers* (MaCs) were started by the City of Malmö and the neighboring communities against the background of a lack of club activities, low school results, and experiences of insecurity among the residents of vulnerable areas due to safety issues. The MaCs are located at schools of different sizes but are otherwise located in different suburbs of Malmö with similar conditions. The MaCs are an investment that the city has made to increase the opportunities for activities in socially unstable areas, where many families have low incomes and cannot afford or otherwise can participate in leisure activities. A starting point in the MaCs is that it is the participants themselves who decide which activities they want, insofar as they are feasible. Children and young people are also invited to help with various activities and some of the younger adults involved become leaders of the activities. It is the participants’ needs and desires that govern the activities available and the MaCs are living laboratories that can develop and rapidly change related to common wishes and needs. Boxing, street dance, football, help with homework, crafts, music, and gymnastics are some of the activities at the MaCs. The MaCs are available in the school premises during afternoons, evenings, and weekends. One goal is that the parents of the children will have a positive image of the school even after school hours.

The areas Hermodsdal, Lindängen, and Apelgården have been classified as three of the most socially deprived areas in Sweden and each of them meets the criteria for being an “*especially vulnerable area*” (7, 24–26). There is a high rate of unemployment and of crime, poor housing, and poor health among the residents, and the levels of education are low (7, 24, 26). Furthermore, child poverty is high, especially in immigrant families (27, 28). Children in *vulnerable populations* can easily face health inequalities (29), affected by their family resources such as housing, employment status, educational opportunities, and other social determinants of health. Migrants and ethnic minorities living in vulnerable city areas are more exposed to stressors and exclusion than other citizens (30). Many children in these areas therefore live in both material and social circumstances that could affect their health, and their voices are seldom raised. It is consequently important that immigrant children living in vulnerable city areas participate in dialogs about health and well-being so that their own perceptions of health can be heard.

## Materials and methods

### Aim

The aim of this study was to investigate the perceptions of health among school-aged children from socially vulnerable areas.

### Design

The study has an explorative mixed-method design with a participatory and inductive approach (31–33) at the MaCs in three of the vulnerable areas in Malmö Municipality. Data is based on focus group interviews, about health and activities, including interviews with school-aged children and youth leaders. The focus was on the children’s own descriptions of health. Data from the MaCs’ own questionnaire is also included.

#### Participant-based perspective

Research with children from a *participatory perspective* has been advocated when school-aged children are active participants in the projects (34). In this study, all children as well as leaders were active participants in the data collection as part of their regular activities at the MaCs.

### Participants

In this study, there were a purposive sampling of participants from three groups, involving either youth leaders or schoolchildren, from each MaC in the three areas.

The first *interview* group consisted of children and the second *interview* group consisted of youth leaders. The inclusion criteria were understanding and speaking Swedish and being interested in sharing their experiences of health and activities.

The third *questionnaire* group of participants consisted of children of all age groups from the three schools involved in the study.

The questionnaires were distributed to children in the schools (*N* = 1,473), by their teachers.

### Data collection

The data collection is based on three datasets: (1) the focus group interviews with children, (2) the focus group interviews with leaders, and (3) the MaCs’ own questionnaire.

Focus groups are an interview form where a small group of committed people talk about a predetermined topic (32). The group participants have something in common (age, gender, experience, etc.) and the question time is led by interviewers/moderators. With the help of the conversation taking place in the group, the perspective on the subject being talked about is broadened, in that the participants can express their opinions while the others listen and complement with their own experiences. The group leaders have an interview guide to help the participants focus on the subject (32). At the end of the meeting, one of the researchers summarizes the conversations and conclusions.

The first dataset, about children’s perceptions of health, consists of *focus group interviews with schoolchildren* accompanied by leaders. After an introduction round, the participants were asked, in pairs, to discuss and write down their conception of health on Post-it^®^ notes. Thereafter, each pair posted their notes on a flipchart and presented their thoughts about health. After each pair had presented their notes and perceptions, the children were asked to group the Post-it^®^ notes into categories making sense of their perception of health. In all three interviews, the children described health in terms of well-being, activity, and participation. Therefore, the rest of the data collection was based on those three themes. Besides being audio recorded, the interviews were documented by the Post-it^®^ notes and by field notes taken by the participating researchers.

The second dataset consists of three *focus group interviews with leaders at the MaCs*. The interviews were scheduled as face-to-face interviews, but because of the Covid-19 pandemic, they were re-scheduled and carried out digitally on Zoom. After an introduction round, the participants were asked to present their experiences about how activities at MaCs can contribute to children’s health, operationalized as well-being, activity, and participation. The interviews were audio recorded and field notes were taken by the participating researcher.

The third dataset consists of the *MaCs’ own questionnaire* about children’s perceptions on activities. The questionnaire contains 15 questions in total and was developed and used by the MaCs to plan their activities for the next semester, and to evaluate the children’s satisfaction with the activities. In the questionnaire, the children reported background data (5 questions) and participation in activities, as well as writing their free comments on the value of different activities and on health, operationalized as well-being, activity, and participation (5 questions). The last five questions are about what the children want to do the next semester (this was not analyzed in this study).

### Data analysis

The *first data analysis* was carried out based on the data from Post-it^®^ notes, recorded interviews, and field notes from focus group interviews with children about health. The analysis used was an inductive content and text analysis (32, 35) to answer the question of children’s perceptions of health. This analysis showed three aspects of health: well-being, activity, and participation.

Thereafter, a *second analysis* was carried out, based on the children’s understanding of the concept of health. In this analysis, data from the first analysis, together with transcribed focus group interviews with leaders as well as the children’s free comments in the questionnaire, were included. This qualitative content analysis was carried out as a deductive content analysis (32) to explore children’s perceptions of health operationalized as well-being, activity, and participation. In this analysis, those three aspects of health, were closely scrutinized and resulted in themes with subthemes.

### Ethical considerations

Ethical research with children and young people is facilitated by a multistep procedure for ensuring that both the research design and protocols are suitable and secure for child participants.

The research group honor the ethical standards set by the Helsinki Committee (36), with respect to autonomy, beneficence, nonmaleficence, and justice (37). On December 1, 2010, the Swedish Parliament approved the government bill “Strategy to strengthen the child’s rights in Sweden” (38). Children should be given the opportunity to express their views on issues that concern them.

All children, parents, and leaders have given their written consent to participate in all research activities in this study.

The study is part of a larger initiative for developing and studying health-promoting activities informed by community-based participatory research in a socially disadvantaged district in the city of Malmö. Ethical approval was granted (Reg.no. 2018/384).

## Results

The first *interview* group consisted of three focus group interviews in total with 21 children aged 10–12 years, all with an immigrant background and the second *interview* group consisted of three focus group interviews in total with 13 leaders (Table 1).

**Table 1:** Participants in the focus group interviews.

Multi-activity Centers (MaCs)	Hermodsdal	Lindängen	Apelgården	In total
Focus group interviews				
Children (*n*=)	2	10	9	21
	2 boys	5 boys	6 boys	13 boys
		5 girls	3 girls	8 girls
(school year) (age)	6	6	5 and 6	5 and 6
	11–12-years	11–12-years	10–12-years	10–12-years
Focus group interviews				
Youth leaders (*n*=)	3	6	4	13
	2 males	3 males	2 males	7 males
(Age)	1 female	3 females	2 females	6 females
	20–30-years	23–42-years	20–30-years	20–42-years

The third *questionnaire* group of participants consisted of children of all age groups from the three schools involved in the study. Almost all children have a migrant background (mainly the Middle East, South America and Asia).

The questionnaires were distributed to children in the schools (*N* = 1,473) and 573 (39%) of the children filled out the questionnaire. Out of the children answering the questionnaire, 69% (61–80%/school) had been actively participating in activities at the MaCs (Table 2).

**Table 2:** Participants answering the questionnaire.

Schools children	Hermodsdal *n* = 523	Lindängen *n* = 620	Apelgården *n* = 330	In total *N* = 1,473
Answered (*n*=)	149 = 28%	196 = 31%	229 = 69%	573 = 39%
Gender				
Boys	84	92	116	292 = 51%
Girls	65	103	111	279 = 49%
Other		1	2	3
School year				
Preschool	20	15	19	
1	29	40	21	
2	18	-	28	
3	22	41	39	
4	21	43	26	
5	19	9	19	
6	12	25	25	
7	8	10	20	
8	-	12	21	
9	–	1	11	
Participated in the multi-activity center				
Yes	119–80.4%	120–61.0%	155–67.9%	394–68.7%
No	30–19.6%	76–38.8%	52–22.8%	158–27.6%
Not answered			21–9.2%	21–3.6%

Four themes emerged after final analysis: *1. Participating in activities increases the child’s sense of well-being*; *2. Participating in activities means having fun together*; *3. Participating in activities is not just being present but having a feeling of involvement*; and *4. Participating in activities creates a sense of faith in the future*. Each of these four themes is presented separately and with quotations from the focus group interviews. In the following text, (Fo gr C) refers to focus groups with children, (Fo gr L) to focus groups with leaders, and (Qu C) to the questionnaire answered by children (Table 3).

**Table 3:** Participating in activities.

Activities in MaCs (participating children)	Hermodsdal *n* = 119	Lindängen *n* = 120	Apelgården *n* = 155	In total (*N*=)
Boy group/Girl group	45	17	86	148
Crafts	20	6	79	105
Baking/Cooking	14	16	50	80
Football	52	18	–	70
Breakfast club	10	1	59	70
School break activity	20	2	40	62
Happy hour	20	12	31	53

### Participating in activities increases the child’s sense of well-being

The result of the analysis shows that participating in activities increases the child’s sense of well-being. The children and leaders described health and well-being as being recognized and feeling good and safe, and having a healthy body.

#### Feeling good

In the focus group interviews, the children pointed out that health and well-being are a *feeling in the body*, a feeling of joy and happiness. The children stressed that the feeling is related to all aspects of the body, physical, psychological, and social. Health and well-being were also described in holistic terms.

“Feel good in the body/Physically and mentally” (Fo gr C)

“I think everything is good” (Qu C)

#### Having a healthy body

In the focus group interviews, the children also pointed out that health and well-being are an *absence of illness and suffering* related to the body. Both in the interviews with children and leaders and in the questionnaire, health and well-being were related to physical body functions in connection with activities and outdoor playing.

“Not being sick/ Not being in pain” (Fo gr C)

“Keep going” (Fo gr L)

“Play outside” (Qu C)

Another aspect brought up was the importance of *healthy living,* like good and healthy food to make the body function well.

#### Feeling safe and recognized

The children talked a lot about the importance of *safety* for health and well-being. Not having to be afraid was considered important, as was not being alone or not being exposed to fights or conflicts among children, adults, and family members.

“Not to be afraid” (Fo gr C)

“That you are never alone/No conflicts” (Qu C)

Children mentioned examples of places or situations that made them feel safe, such as participating in the girls’ group.

“In a girl group it has been safe to just be with girls to avoid all the noisy boys” (Qu C)

To have a feeling of health and experience well-being, the children wanted to be seen and *recognized* by the leaders at the MaC. Also, the importance for the child to be recognized in the family at home was mentioned.

“Be there for the child/Pay attention to how you feel at home” (Fo gr L)

Having a feeling of health and well-being by being recognized, was often described in terms of small talk or inclusion. But those small recognitions were described as leading to close relations and a feeling of trust.

“Just hang out” (Qu C)

“Relation/Trust” (Fo gr L)

### Participating in activities means having fun together

The result of the analysis shows that children describe participating in activities and having fun as an important agent for health, when doing things together with friends and leaders.

In the interviews and in the questionnaire, the children saw health as being related to different *activities*. The most frequent activities the children participated in were divided groups for boys and girls, different kinds of crafts, sports activities, such as football, and sports combined with cooking or baking.

“Football, baking/cooking, boys’ group, girls’ group, crafts, games & gaming” (Qu C)

#### Having fun

The children expressed that some activities were *more fun* and popular to participate in, such as:

“Baking and that we have baked good cakes” (Qu C)

“Crafts; to paint, we have beaded, make slime, paint gym bag and backpacks” (Qu C)

“Play football” (Qu C)

“Boy group because we play games and watch movies” (Qu C)

But the preferences changed in relation to what was offered, and in relation to age, gender, and, mainly, friends’ choices.

The children also pointed out that there were *activities they wanted to have more of*. Those activities were often the ones where they needed to leave the neighborhood or had to pay an entrance fee, such as:

“Excursions/Go-kart” (Fo gr C)

For an activity to lead to a feeling of health the *activity needed to be fun*. Talking about different activities, the children often stated that they were fun. But they also expressed a feeling of participating in a good activity, leading to *emotional expressions.*

“I think it’s fun/It was good/Laugh” (Qu C)

“Be happy” (Fo gr C)

The leaders, in turn, mentioned the importance of the children wanting to be present:

“They want to go/They come” (Fo gr L)

### Doing things together

Children and leaders stressed the importance of giving children the possibility of doing the *activities together* with others, such as friends and adults. The children commented that activities at the MaCs gave them opportunities to *meet their friends*. Moreover, participating in activities gave the children a chance to *make new friends*.

“You have an activity in your spare time and can hang out with friends at the same time” (Qu C)

“What has been good is that I had fun with friends and got many new ones” (Qu C)

Participating in activities also created a good base for *good relationships*. Both children and leaders stated the importance of a good atmosphere and of *trustful relationships* between children and leaders. The activities were especially valued by children with few friends.

“The staff are very nice and fun to hang out with” (Qu C)

“Oh, continue to have such a positive spirit every day” (Qu C)

“Long-term relationships” (Fo gr L)

“Leaders and activities are important for a child who does not have friends” (Fo gr L)

Furthermore, the leaders emphasized the value of being present and available for establishing a trustful relationship and of *spending time with the children*.

“Attendance/Time to see children” (Fo gr L)

### Participating in activities is not just being present but having a feeling of involvement

The result of the analysis shows that participating in activities was described by children and leaders as not just being present but having a feeling of involvement, by being able to have an influence and to participate on one’s own terms.

#### Being able to influence

For the children, it was important to be able to be actively involved and to be *able to influence* their own situation, with regard to the activities they were participating in. The children described how it was fun to be part of the decision-making. The leaders described how they always invite the children to be part of the planning of activities for the children to be able to make choices.

“We choose what to do at the activities/That it was fun and that we get to decide” (Qu C)

“The child is involved in planning” (Fo gr L)

But at the same time, the children stated that they wanted to have a greater influence. The children gave examples of aspects and situations they *wanted to improve*, but where their wishes were not being met. The aspects that the children most often mentioned as needing to be improved were the possibilities to choose regarding the activities, both the length, and the design of an activity. Most suggestions for improvements can be considered to be wishes for activities, often the ones requiring traveling and being expensive.

“What I think we can improve is to do some more things we want and get to decide a little more” (Qu C)

“Have longer activities/Go to fun and different places” (Qu C)

“Water-fight swim at Lindängsbadet [an outdoor swimming pool]” (Qu C)

“I want more excursions with our activities with the girl group, then we can go out on the town or go to Tosselilla [an amusement park],” a Zoo or just a café (Qu C)

#### Participating on one’s own terms

Both children and leaders pointed out the importance of activities at the MaC being a complement to activities in school, not being compulsory, competitive, or requiring achievements. The children commented that they appreciated the availability of the activities. The importance of letting children *be themselves* was also stressed.

“The opposite of school/Voluntary/Not achievement” (Fo gr L)

“It has also been good that it’s after school” (Qu C)

“The children can come as they are/Unconditional” (Fo gr L)

Moreover, the leaders highlighted the importance of having activities that make all children *participate equally*, regardless of their family’s culture, religion, or economic situation.

“The families who have no money, the child can still take part in activities” (Fo gr L)

### Participating in activities creates a sense of faith in the future.

The result of the analysis shows that children and leaders describe participating in activities as a way for the child to gain faith in the future and to learn by trying new things, thus positively influencing the child’s development, and resulting in increased self-confidence and a positive behavior.

#### Learning new things

Both the children and the leaders made statements about how important it is to be able to gain knowledge. The knowledge was gained by trying new activities and by engagement on the part of the leaders. Also, trying new activities could help children find new interests.

“Gives me a lot of knowledge/That the leaders help us, teach us new things” (Qu C)

“You try new things, which makes you find new interests” (Qu C)

Trying new activities can also increase the child’s creative side. For children, finding new interests might even lead to a life-long interest or career.

“Creativity in music, art, and crafts” (Fo gr L)

“Find geniuses” (Fo gr L)

#### Positively influencing the child’s development

During the focus group interviews with the leaders, they drew attention to the possibility to positively influence the child’s development. Participating in activities and relationships with children and leaders, can influence the child toward a *changed behavior* if needed. Moreover, the relationship, built on mutual trust between children and leaders, can create more *self-confidence* in children. Being pointed out as a good friend was also an activity that accelerated the child’s self-confidence.

“Friend of the week/Influence the child/Positive reinforcement/Change behavior” (Fo gr L)

“Long-term relationship” (Fo gr L)

Furthermore, the leaders described *meaningful activities* as a way of creating hope for the future. Meaningful activities could be the joy of discovering, such as making crafts to take home and show to parents and siblings or creating.

“Discover/Create/Creation joy” (Fo gr L)

“Have something to take home and show” (Fo gr L)

## Strengths and limitations

### Discussion of the method

Conducting focus groups is a method for reducing the power imbalance between researcher and children (39, 40). The children in the present study thought that taking part in focus group interviews was fun, which suggests that the children’s perspective on health was represented. However, the children’s perceptions of health may have been influenced by interviews being undertaken at the MaCs, and by having a few leaders present at the interviews. The Post-it^®^ notes were used as a starting point for the focus group discussions, and the children could present an unlimited number of notes and then sort and discuss the ones of their choice in the focus groups. This gave the children freedom to experiment through play, which they pointed out at the end of the study. Moreover, the Post-it^®^ notes opened for reflections on aspects that were not always obvious to an adult. The smaller number of participating children as well as leaders, in some of the focus groups, is a possible limitation. But it might be easier for children in a focus group to stay focused and be heard when there are only few participants in the group (40, 41).

Another possible limitation is the questionnaire, which were developed for use by the MaCs themselves. The questions did not pass through a scientific screening before being used. Therefore only a few questions were analyzed, such as frequencies of participation, together with the free comments written by children. The authors had no control over how many children were in fact reached by the questionnaire, and no knowledge of other reasons for not participating. Also, the actual response rate of the questionnaire is not known to the authors, and there is no data on those who did not respond, hence the interpretation of data needs to be done cautiously. Short comments and short answers in interviews are not uncommon in studies with children (42) but can be a problem in the analysis. Still, a short comment from a child could contain valuable information on the subject under study (40, 41, 43).

Finally, the transferability of the results may be questioned because this study is limited to immigrant children from vulnerable areas in Sweden.

### Discussion of the results

The result of this study shows that for the children health could be described in terms of different dimensions, such as well-being, activity, and participation. This could be related to the findings of Natapoff (42) and Almquist et al. (12). When they interviewed young children about their concept of health, they concluded that ***overall health*** seems to be an abstract concept that is multi-dimensional and hard to define for children and that children’s perception of health involves both physical and mental dimensions.

An important finding in this study was that the children related health to participation, in a supportive environment, where they could perform ***activities*** based upon their own choices. This is consistent with other studies that connect health and ***well-being*** for children to participating in activities (13, 44). Participation in activities also seems to influence increased self-rated health, life satisfaction, and happiness (45) Moreover, Lloyd and Emerson (46) found a correlation between children’s subjective well-being and their perceptions that their participation rights are respected.

The children and leaders in this study described health and well-being as, among other things, having a healthy body. However, the cost may restrict families’ possibilities to adopt ***healthy behaviors*** such as eating fruit and vegetables (47–49). Unhealthy behavior regarding nutrition can affect school results negatively and be a risk for children’s physical and mental well-being, so regular lunches in school are very important (50). The children who participated in this study pointed out the value of free meals, when participating in the Breakfast club and in Baking and Cooking activities.

Moreover, in the present study, children pinpointed participating in activities as an important means for health and enjoying oneself. They argued that activities are highly related to being able to do things together with friends and young leaders. ***Positive social interactions*** with friends and adults give children social skills that will be important for them later in life (51), as well as happiness and self-esteem (52, 53), and may also have a long-term effect on social adjustment (54). It is well known that social support and activities are important for a child’s development and for how they experience and understand their own self (55). School-aged children have described leisure activities as joint and meaningful activities (14) and having fun and feeling good have been shown to be children’s reasons for participation in activities (56).

Another aspect that the children and leaders in this study particularly pointed out was the importance of creating a good and ***trustful relationship*** between the children and the leaders, to promote children’s engagement in activities. This process of trust requires recognizing and respecting the child and paying attention to the child as an individual as well as being familiar with the child’s family (57, 58). In this study, the most frequent activities the children were involved in were single-sex group activities, that is, girls’ and boys’ groups. Possibly the main reason for choosing those groups was the content of the activity and the close relation to the leaders. But the single-sex approach also attracted the children. Single-sex school programs are under discussion, regarding both advantages and disadvantages [see, e.g., Debating Europe, 2021 (59)], but after-school activity has not been evaluated as frequently. In this study, the high number of girls participating in the activities might be a result of non-gender-mixed activities, since gender-mixed activities sometimes prevent children from migrant families from participating (60). Hence, the first goal is to get children to participate, the next goal is to counteract discrimination.

Furthermore, the children gave examples of ***activities they enjoyed*** and found fun, and such activities have several positive effects on children’s development (13). Among other activities in this study, the children engaged in physical activities, such as football and dance, which was, by the children, mentioned as joyful and important for healthy living. It is well known that reducing a child’s possibilities to have an active lifestyle may in the future lead to an increase in chronic diseases (61) and health-related quality of life (62).

Yet, children’s needs are not met by simply having playgrounds for football and a schoolyard; children need to explore and discover different spaces, such as their neighborhood, the city, and the surrounding landscape (3, 61). This was illustrated in the present study by the fact that the children wished for more ***outdoor activities and excursions***. Research has shown that exploring the physical environment is important for children’s development of self (63), and that nature activities could benefit children’s mental well-being (64). In our earlier study on children’s perception of their outdoor environment in a socially vulnerable area (65), the children pointed out places where they must not go, adults having warned them about the risks. In this way the adults restricted the children’s freedom to move around in the local environment. The local environment, as part of the ecological systems (66, 67), is important for the children’s lives in terms of socialization, health, and development. Eriksson and Dahlblom (44) show a close link between social activities and physical environment. They argue that children’s access to places for health promotion, such as outdoor arenas for activities, constitutes an important social capital reducing inequalities between different neighborhoods.

Furthermore, children living in vulnerable areas have been shown to be less engaged in ***organized activities*** (19). Not taking part in organized activities might result in lower achievement in school work and affect mental health (50). Results of an evaluation from an adult’s perspective in 2015 (68) show that most children think it is fun to come to the MaC, that children feel seen and listened to, and that they feel very proud of their work. Another result shows that the school staff describe the school environment as more positive since the start of the MaCs, in that the atmosphere at the school becomes calmer, and the children feel safer and can concentrate better. This can be measured by the calmness during the lessons, less noise, fewer false fire alarms, and reduced destruction costs, as well as lower stress for the school staff. Yet another clear result is that, in one of the areas, the grades of the students in school year nine have risen by 11 percent after the start of the MaC (68).The result of this study shows that ***participating*** in activities was described by children and leaders as not just being present but having a feeling of involvement, by being able to have an influence and to participate on one’s own terms in organizing the activities. The WHO’s (51) definition of participation as “an individual’s engagement in his or her life situation” considers both the social and functional aspects of health, with the individual as an active participant in their own development. Eriksson and Granlund (69) define participation as “a feeling of belonging and engagement experienced by the individual in relation to being active in a certain context,” divided into two dimensions: presence (i.e., physically being there) and engagement (i.e., expressions of involvement) (70), which is in line with what the children in this study have described.

In the present study, the children and the leaders pointed out the importance of involving children in all ***decision-making*** regarding both the choice and the outline of activities at the MaC. Many documents about human rights have drawn attention to the right for children to participate in decision-making processes. However, the implementation of this right through meaningful participation has been a challenge (71, 72), even if it is now accepted in research and policy making (72).

The children in this study were somewhat disappointed at not being part of the decision-making regarding the ***choice of all activities***. Some suggestions for activities were related to a need to travel or were expensive. As most children in this study come from disadvantaged families, the leaders stated that those families might not be able to offer such activities themselves. Thus, despite the wish for children to be able to participate on equal terms in activities, independently of family economy, cost may restrict families’ opportunities to participate in fee-based activities (73, 74).

Another important result of this study shows that children and leaders describe participating in activities as a way for the child to ***gain faith in the future*** and to learn by trying new things, thus positively influencing the child’s development, and resulting in increased self-confidence and a positive behavior. Health inequalities can also arise because children living in disadvantaged families are exposed to psychosocial stress (75). But positive development and positive behaviors can be gained by relationships and activities fostering knowledge, competence, confidence, character, and a feeling of contributing. Children’s participation and behaviors are important for the direction of their future development (55). Today children can be seen as active citizens. Their possibility for participating in activities allows them to actively understand what promotes their health and well-being. When children participate in organizing meaningful activities together, this also contributes to their understanding of both self and the society.

## Conclusion

This study contributes to an increased knowledge of children’s own perceptions of health and of the organization of activities that can promote health, well-being, participation, and a meaningful life. Healthy activities should not be viewed as isolated islands; they are integrated into the society in a system consisting of both of social relations and the material environment (76).

The result of this study shows that participating in activities increases the child’s sense of well-being. Moreover, participating in activities in the MaCs, as well as in organizing those activities, allowed the children in the present study to actively understand what contributes to their health and well-being, which may also further a sense of connectedness, motivation, and understanding of both themselves and society.

This implies that child participation is important in relation to health interventions aimed at attempting to change children’s health behavior. Also, knowledge about the variations in children’s perceptions of health can help researchers and professionals in defining relevant outcomes in terms of such interventions. Based upon the results of this study, the research team will continue to work in participation with the children and the youth leaders, to form the basis for the implementation of intervention efforts on a larger scale, in other cities in Sweden.

## Data availability statement

The raw data supporting the conclusions of this article will be made available by the authors, without undue reservation.

## Ethics statement

The studies involving human participants were reviewed and approved by the Ethical Review Board in Lund (Reg. no. 2018/384). Written informed consent to participate in this study was provided by the participants’ legal guardian/next of kin.

## Author contributions

GI, MR, and KE participated in the design of the study, also participated one or more of the focus groups and in the analysis process, and wrote the manuscript. All authors contributed to the article and approved the submitted version.

## Funding

The current study was carried out as a part of “The Health Promotion Platform in Collaboration” at Malmö University. The platform was funded by VINNOVA from 2016 to 2019 (Reg. no. 2016-00421, 2017-01272), and by the Faculty of Health and Society at Malmö University from 2020 to 2021. The publication fee was funded by a publication grant by Malmö University.

## Conflict of interest

The authors declare that the research was conducted in the absence of any commercial or financial relationships that could be construed as a potential conflict of interest.

## Publisher’s note

All claims expressed in this article are solely those of the authors and do not necessarily represent those of their affiliated organizations, or those of the publisher, the editors and the reviewers. Any product that may be evaluated in this article, or claim that may be made by its manufacturer, is not guaranteed or endorsed by the publisher.
